# The return of spermatogenesis in transgender women ceasing gender-affirming hormone therapy

**DOI:** 10.1016/j.xcrm.2022.100835

**Published:** 2023-01-17

**Authors:** Mabel Yau, Joshua D. Safer

**Affiliations:** 1 Mount Sinai Center for Transgender Medicine and Surgery, New York, NY, USA; 2 Division of Pediatric Endocrinology, Mount Sinai Kravis Children’s Hospital, New York, NY, USA; 3 Division of Endocrinology, Mount Sinai Health System and Icahn School of Medicine at Mount Sinai, New York, NY, USA

## Abstract

Fertility has become a priority in transgender health research. In this issue of *Cell Reports Medicine*, a study by de Nie et al.^1^ of nine transgender women demonstrates sperm production after the cessation of gender-affirming hormone therapy (GAHT). Their results suggest the transient nature of gonadal suppression by GAHT.

## Main text

Fertility is a patient-centered outcome of transgender care that has become a priority of health research. Many transgender patients seek gender-affirming hormone therapy (GAHT). In transgender women and other transfeminine individuals, GAHT usually involves estrogen, testosterone blockers, and occasionally progesterone. Before transgender women begin GAHT, clinical guidelines encourage sperm cryopreservation to preserve fertility.^2^ Estrogen therapy suppresses the hypothalamic-pituitary-gonadal (HPG) axis. Through this negative feedback, testosterone production and associated masculinizing characteristics are reduced.^3^ GAHT is also associated with reduced spermatogenesis, leading to impaired fertility.^4^ The HPG axis coordinates essential testicular functions needed for spermatogenesis through the actions of follicle-stimulating hormone and luteinizing hormone as well as the maintenance of high intratesticular testosterone concentration.^5^ Low serum testosterone levels are associated with low sperm maturation rates.^6^ Studies in transgender women have shown reduced or absent sperm production at the time of orchiectomy (a form of gender-affirming surgery) in individuals who were on GAHT or shortly after discontinuation.^6^^,^^7^ A recent growing awareness, particularly in the non-medical public, of the role that GAHT plays in the care of trans patients has triggered a concern that GAHT could lead to an irreversible suppression of gonadal reproductive function.

Many trans people do not wish to restore gonadal reproductive function—but some might. In this issue of *Cell Reports Medicine,* de Nie et al. report a small case series of nine transgender women who stopped GAHT and demonstrated renewed sperm production.^1^ In this small cohort, four individuals conceived naturally with their current partners after GAHT cessation. These results suggest a possibly transient nature to GAHT-induced gonadal suppression and the potential to restore fertility after GAHT cessation. This is a promising finding for those who choose to have biological children later in life but either initially declined fertility preservation or wish to conceive children without assisted reproduction.

The time to return of spermatogenesis varied in this group. Viable sperm were identified as early as 3 months after the cessation of GAHT. One individual required a testicular biopsy to extract spermatozoa after 17 months. The authors report no obvious relationship between the duration of GAHT and the time to first identifyspermatozoa. However, the sample size was small, and the timing of semen collection varied. Low serum testosterone levels are associated with low sperm maturation rates. Thus, the rise in serum testosterone levels along with gonadotropin levels may be associated with the resumption of spermatogenesis. Larger studies with stricter baselines and in-study assessments of spermatozoa levels, longitudinal follow up, and correlation with hormonal levels are needed to determine the clinical factors associated with an earlier return of spermatogenesis and the hormonal predictors of spermatogenesis. Also, semen analyses were not performed before or during GAHT in this study. Other studies of transgender women before the initiation of GAHT show that they had a higher proportion of sperm abnormalities compared with non-screened cisgender males.^4^^,^^8^ de Nie et al. have previously reported decreased semen quality in a large cohort of which only a small proportion had received GAHT at the time of fertility preservation.^9^^,^^10^ They identified behavioral factors, including wearing tight undergarments and extensive “tucking” (a practice of some trans women and gender-conforming individuals to minimize the external contour of their genitals), that negatively impacted spermatogenesis.^9^^,^^10^ However, only a small proportion reported performing these habits. Behaviors and medical histories did not explain the overall rates of infertility they found.^10^ Part of the etiology of this apparent increased rate of baseline infertility remains unclear. Understanding such phenomena will continue to improve the care and counseling of transgender women.

Even with their limitations, the results reported by de Nie et al. are encouraging,^1^ but they do not obviate the need for counseling prior to the initiation of therapy. Clinically, we observe that many transgender women and transfeminine people decline to suspend GAHT for fertility even if there were a chance that such a maneuver could successfully restore spermatogenesis. In summary, de Nie et al. demonstrate an encouraging result that suggests that GAHT-induced gonadal suppression might be reversible.

## References

[1] de Nie I., van Mello N.M., Vlahakis E., Cooper C., Peri A., den Heijer M., Meibner A., Huirne J., Pang K.C. (2022). Successful restoration of spermatogenesis following gender-affirming hormone therapy in transgender women. Cell Rep. Med.

[2] Hembree W.C., Cohen-Kettenis P.T., Gooren L., Hannema S.E., Meyer W.J., Murad M., Rosenthal S.M., Safer J.D., Tangpricha V., T'Sjoen G.G. (2017). Endocrine treatment of gender-dysphoric/gender-incongruent persons: An endocrine society clinical practice guideline. Endocr. Pract..

[3] Collet S., Gieles N., Wiepjes C.M., Heijboer A.C., Reyns T., Fiers T., Lapauw B., den Heijer M., T'Sjoen G. (2022). Changes in serum testosterone and adrenal androgen levels in transgender women with and without gonadectomy. J. Clin. Endocrinol. Metab.

[4] Rodriguez-Wallberg K.A., Haljestig J., Arver S., Johansson A.L.V., Lundberg F.E. (2021). Sperm quality in transgender women before or after gender affirming hormone therapy-A prospective cohort study. Andrology.

[5] Anderson R.A., Baird D.T. (2002). Male contraception. Endocr. Rev.

[6] Vereecke G., Defreyne J., Van Saen D., Collet S., Van Dorpe J., T'Sjoen G., Goossens E. (2021). Characterisation of testicular function and spermatogenesis in transgender women. Hum. Reprod.

[7] Jiang D.D., Swenson E., Mason M., Turner K.R., Dugi D.D., Hedges J.C., Hecht S.L. (2019). Effects of estrogen on spermatogenesis in transgender women. Urology.

[8] Amir H., Perl L., Barda S., Lantsberg D., Becker A.S., Israeli G., Azem F., Oren A. (2022). Adolescent transgender females present impaired semen quality that is suitable for intracytoplasmic sperm injection even before initiating gender-affirming hormone treatment. Reprod. Sci.

[9] de Nie I., Meißner A., Kostelijk E.H., Soufan A.T., Voorn-de Warem I.A.C., den Heijer M., Huirne J., van Mello N.M. (2020). Impaired semen quality in trans women: Prevalence and determinants. Hum. Reprod.

[10] de Nie I., Asseler J., Meißner A., Voorn-de Warem I.A., Kostelijk E.H., den Heijer M., Huirne J., van Mello N.M. (2022). A cohort study on factors impairing semen quality in transgender women. Am. J. Obstet. Gynecol.

